# Childhood-Onset ANCA- Associated Vasculitis: single center experience from Central California

**DOI:** 10.1186/s12969-023-00853-4

**Published:** 2023-07-03

**Authors:** Deepika Singh, Sukesh Sukumaran

**Affiliations:** Division of Pediatric Rheumatology, Department of Pediatrics, Valley Children’s Healthcare, Madera, CA USA

**Keywords:** ANCA associated vasculitis, Microscopic polyangiitis, granulomatosis with Polyangiitis, Eosinophilic granulomatosis with polyangiitis, Vasculitis, Small vessel vasculitis

## Abstract

**Background:**

Childhood-onset ANCA-associated vasculitides (AAV) are characterized by necrotizing inflammation and include granulomatosis with polyangiitis (GPA), microscopic polyangiitis (MPA), and eosinophilic granulomatosis with polyangiitis (EGPA). Pediatric data is scare and there have been no prior studies examining the characteristics of pediatric AAV in Central California.

**Methods:**

This retrospective study comprised AAV patients ≤18 years of age, diagnosed between 2010 and 2021, in Central California. We analyzed initial presentation including demographics, clinical, laboratory characteristics, treatment, and initial outcomes.

**Results:**

Of 21 patients with AAV, 12 were categorized as MPA and 9 with GPA. Median age at diagnosis was 13.7 years in MPA cohort and 14 years in GPA. MPA cohort were majority females (92% versus 44%). 57% of the cohort were racial/ethnic minority including Hispanics (n = 9), Asians (n = 2), multiracial (n = 1) and 43% were white (n = 9). MPA patients were more frequently Hispanic (67%), meanwhile GPA patients were frequently white (78%). Median duration of symptoms prior to diagnosis was 14 days in MPA cohort and 21 days in GPA cohort. Renal involvement was frequent (100% in MPA and 78% in GPA). GPA cohort had frequent ear, nose and throat (ENT) involvement (89%). All patients were ANCA positive. All Hispanic patients were MPO positive, meanwhile 89% of white patients were PR3 positive. MPA cohort tended towards more severe disease with 67% requiring ICU admission and 50% requiring dialysis. Two deaths were reported in MPA cohort, related to Aspergillus pneumonia and pulmonary hemorrhage. In MPA cohort, 42% received cyclophosphamide in combination with steroids and 42% received rituximab in combination with steroids. GPA patients received cyclophosphamide, either with steroids alone (78%) or in combination with steroids and rituximab (22%).

**Conclusions:**

Microscopic polyangiitis was the most frequent AAV subtype with female preponderance, shorter duration of symptoms at onset and higher proportion of racial/ ethnic minority patients. Hispanic children demonstrated frequent MPO positivity. Trends towards higher rates of ICU requirement and need for dialysis upon initial presentation was noted in MPA. Patients with MPA received rituximab more frequently. Future prospective studies are needed to understand differences in presentation and outcomes in childhood onset AAV between diverse racial-ethnic groups.

## Introduction

Anti-neutrophil cytoplasmic antibody (ANCA)-associated vasculitides (AAV) are a group of systemic vasculitides characterized by necrotizing inflammation predominantly affecting the small blood vessels. They include Granulomatosis with Polyangiitis (GPA), Microscopic Polyangiitis (MPA), and Eosinophilic Granulomatosis with Polyangiitis (EGPA) [1]. AAV are life-threatening diseases that are associated with significant morbidity and mortality [2–4].

Childhood-onset ANCA-associated vasculitis is extremely rare and data on this entity is sparse. Increasing rates of incidence of childhood AAV has been demonstrated [5, 6], similar to observation from adult studies [7, 8]. Most of the existing literature on childhood onset AAV is derived from Europe and North America with predominantly white patients [5, 9, 10]. Studies in adults with AAV have reported variability in the clinical phenotype of disease between various ethnic and racial backgrounds [11]. Differences in incidence and prevalence of GPA and MPA based on geographical location have been noted in studies from China [12], Latin America [13, 14], Japan and UK [15]. More recent adult studies from AAV within United States have demonstrated differences in disease and damage indices in Hispanics in comparison to Caucasians [16, 17].

Gaining an understanding of disease presentation and complications of pediatric AAV in multiethnic populations may help identify gaps in the evaluation and treatment of children with AAV. This is especially important considering that genetic susceptibility and environmental risk factors all contribute to the risk of developing AAV [1, 18]. To date, there have been no studies examining the characteristics of childhood- onset ANCA associated vasculitis in ethnically diverse Central California. California’s Central Valley includes 9 counties among which Fresno, Kern and Tulare counties are the largest. Fresno has a Hispanic majority population of 54.7%, Kern has 56.1% and Tulare has the highest with 66.7% [19–21]. These percentages surpass the average Hispanic population in California, which is 40% according to US census data [22].

Our objective was to analyze the demographic, clinical characteristics, laboratory manifestations, and initial outcomes of children diagnosed with ANCA associated vasculitis from 2010 to 2021 who resided in Central California.

## Methods

### Study design

We performed a retrospective case review of patients ≤18 years of age diagnosed with AAV at Valley Children’s Hospital, the only freestanding tertiary care children’s hospital in Central California, with a service area encompassing 1.3 million children. We identified cases between January 1, 2010 to March 31, 2021 from electronic health records using International Classification of Diseases-9 and International Classification of Diseases-10 codes for ANCA vasculitis. The study was approved by the Valley Children’s Healthcare institutional review board.

We included patients ≤18 years of age who met the diagnosis of AAV (i.e., GPA, MPA, or EGPA) based on European Alliance of Associations for Rheumatology (EULAR) /Pediatric Rheumatology International Trials Organization (PRINTO)/ Pediatric Rheumatology European Society (PReS) criteria for diagnosis of granulomatosis with polyangiitis [23]. The diagnosis of GPA was defined by presence of 3 of the 6 features including (1) histopathology (granulomatous inflammation on biopsy) (2) upper airway involvement (chronic purulent or bloody nasal discharge or recurrent epistaxis/crusts/granulomata) (3) laryngotracheal bronchial involvement (subglottic, tracheal or bronchial stenosis) (4) pulmonary involvement (alveolar hemorrhage, presence of nodules, cavities or fixed pulmonary infiltrates on chest radiograph or CT scan) (5) renal involvement (proteinuria, hematuria or necrotizing pauci-immune glomerulonephritis). Patients with positive ANCA and either renal limited vasculitis or clinical presentation compatible with AAV without fulfilling EULAR/PReS criteria were classified as MPA using algorithm by Watts et al. [24]. Patients > 18 years of age were excluded from the study. Race/ethnicity was determined by self-report.

Demographic and clinical data including initial clinical presentation, duration of symptoms prior to diagnosis, insurance status, postal zip code of residence, laboratory parameters, histopathology and radiological investigations, treatment and outcomes including length of hospital stay and mortality were collected from electronic medical records. All patients were diagnosed during inpatient hospitalization. We did not find any patients diagnosed as outpatient which is plausible given the morbidity associated with AAV. Kidney biopsy results were classified as focal, crescentic, sclerotic or mixed according to histopathologic classification of ANCA-Associated Glomerulonephritis published in 2010 by Berden et al. [25]. Samples with ≥ 50% normal glomeruli were classified as focal; those with ≥ 50% cellular crescents as crescentic, those with ≥ 50% sclerotic glomeruli as sclerotic and those with a combination of normal, crescentic and sclerotic glomeruli, and all occurring in < 50% of glomeruli as mixed. Given that disease activity scores were not available, we used rates of intensive care unit (ICU) admission, length of hospital stay, mortality and need for dialysis and plasmapheresis, as surrogate markers of severe disease.

Continuous data were expressed as a median and interquartile range, categorical data as frequency and percentages. For comparison of categorical data, Chi-square test was performed. Fisher exact test was used when expected frequency was less than 5. For comparison between continuous variables, Mann-Whitney U tests was used. A *p* value of ≤ 0.05 was considered statistically significant.

## Results

### Demographics, clinical and laboratory findings

**Fig. 1:**
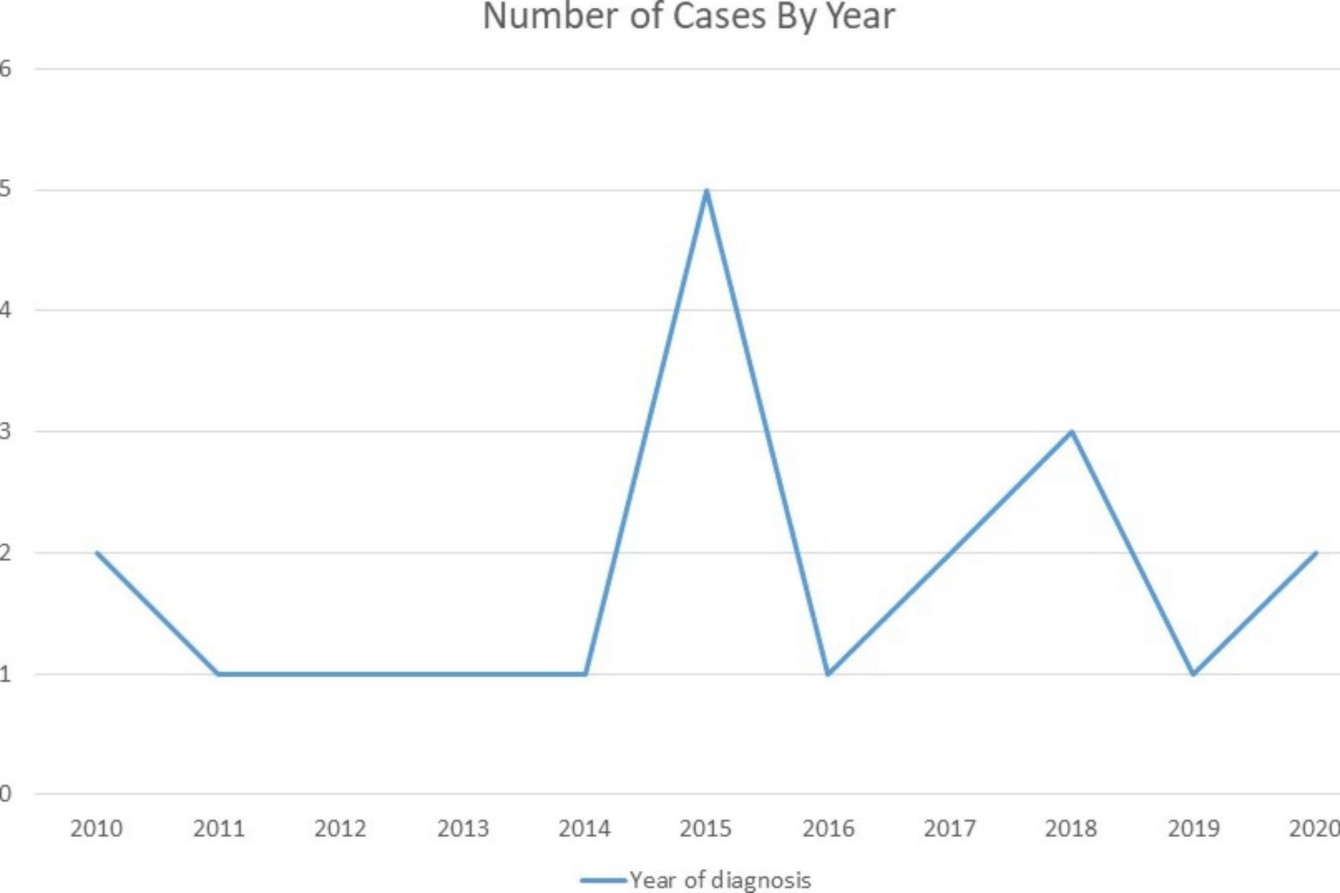
Case distribution by year of diagnosis

We identified 21 patients who were diagnosed with AAV. Baseline demographics are summarized in Table 1. Over half of the 21 cases were diagnosed from 2015 to 2018 (Fig. 1). Twelve patients (57%) in the cohort were diagnosed with MPA and nine (43%) with GPA (Table 1). None of AAV patients in our cohort had EGPA. The median age at diagnosis was 14.0 years (IQR 12.2–15.6). Median age at diagnosis in MPA cohort was 13.7 years (10.4–15.5) and 14 years (IQR 12.15–15.64) in GPA (Table 1). We found a significantly higher proportion of females within the MPA cohort (92%) in comparison to GPA (44%), p = 0.05. Median duration from reported onset of symptoms to initial hospitalization was 14 days (IQR 7–28) in MPA cohort and 21 days (IQR 21–42) in GPA cohort but this was not statistically significant (p = 0.2).

**Table 1:** Demographic characteristics of patients with childhood ANCA-associated vasculitis

	Total N = 21	MPA N = 12	GPA N = 9	p-value(*)
Median age at diagnosis (years, IQR)	14.0 (12.2–15.6)	13.7 (10.4–15.5)	14 (13.1–16.7)	0.2
Females (n, %)	15 (71%)	11 (92%)	4 (44%)	0.05
Hispanic	9 (43%)	8 (67%)	1 (11%)	0.01
Asian	2 (9%)	1 (8%)	1 (11%)	0.8
Multiracial	1 (5%)	1 (8%)	0 (0%)	1
White	9 (43%)	2 (17%)	7 (78%)	< 0.01
Median duration of symptoms (days, IQR)	21 (14–28)	14 (7–28)	21 (21–42)	0.2

IQR = Interquartile range, PR-3 = Proteinase 3, MPO = Myeloperoxidase, *MPA versus GPA

Table 2 describes clinical characteristics including organ system manifestations at the time of disease presentation, lab characteristics including creatinine, ANCA positivity and biopsy. Constitutional symptoms including fever, malaise, fatigue, or weight loss were present in all patients. Renal involvement was seen in 19/21 (90.4%) with hematuria, proteinuria, hypertension, glomerulonephritis on renal biopsy and/or elevated creatinine (Table 2). All patients in the MPA cohort had renal involvement at presentation. In comparison, 77.7% in GPA cohort demonstrated renal involvement (p = 0.17). Median peak creatinine was higher in MPA cohort at 7.4 mg/dL (IQR 1.3–14.1), compared to 2.4 mg/dL (IQR 0.6–6.2) in GPA cohort (p = 0.2).

**Table 2:** Clinical features at presentation in patients with childhood ANCA-associated vasculitis

	Total N = 21	MPA N = 12	GPA N = 9	p-value (**)
Systemic	21 (100%)	12 (100%)	9 (100%)	1
Cutaneous	7 (33%)	3 (25%)	4 (44%)	0.4
*Petechiae/purpura*	4	1	3	
*Rash*	3	2	1	
ENT	9 (43%)	1 (8%)	8 (89%)	< 0.01
*Epistaxis*	3	0	3	
*Sinusitis*	8	1	7	
*Chronic nasal discharge*	5	0	5	
*Subglottic involvement*	1	0	1	
*Otitis/mastoiditis*	2	0	2	
ENT Biopsy	3 (14%)	0 (0%)	3 (33%)	0.06
Pulmonary	16 (76%)	8 (67%)	8 (89%)	0.33
*Alveolar hemorrhage/hemoptysis*	6	3	3	
*Respiratory failure*	3	2	1	
*Pleural effusion*	4	2	2	
*Pulmonary nodules*	6	0	6	
*Pulmonary infiltrates*	11	7	4	
*Cavitary lesions*	4	0	4	
Renal impairment	19 (90%)	12 (100%)	7 (78%)	0.17
*Hematuria*	16	10	6	
*Proteinuria*	16	9	7	
*Hypertension*	8	6	2	
Creatinine at presentation, mg/dL (median, IQR)	1.56 (0.75–11.5)	6.6 (1.1–14.1)	1 (0.6–2.9)	0.16
Peak creatinine, mg/dL (median, IQR)	2.93 (0.75–11.5)	7.4 (1.3–14.1)	2.4 (0.6–6.2)	0.2
Renal Biopsy	16 (76%)	10 (83%)	6 (67%)	0.6
GI	6 (29%)	2 (17%)	4(44%)	0.33
*Abdominal pain*	6	2	4	
Cardiovascular	4 (19%)	3 (25%)	1 (11%)	0.60
*Pericarditis*	4	3	1	
*Cardiomegaly*	1	1	0	
Nervous	4 (19%)	1 (8%)	3 (33%)	0.27
*Facial paralysis*	1	0	1	
*Mononeuritis*	2	0	2	
*Headache*	1	1	0	
Anti-MPO positive (n, %)	12 (57%)	10 (83%)	2 (22%)	< 0.01
Anti-PR-3 positive (n, %)	10 (48%)	1 (8%)	9 (100%)	< 0.0001
Hemoglobin, g/dL (median, IQR)	8.9 (5.8–10.4)	6.9 (5.3-9)	10.4 (9-11.2)	0.03

IQR = Interquartile range, PR-3 = Proteinase 3, MPO = Myeloperoxidase, **MPA versus GPA

Renal biopsy was obtained in 76% (16) of subjects. Sclerotic disease was seen in 4 out of 16 biopsies. MPA patients frequently had sclerotic (40%) and mixed (30%) subtypes, meanwhile in GPA cohort, crescentic subtype (67%) was most frequent (Table 3).

**Table 3:** Renal histopathology according to histopathologic classification of ANCA- associated glomerulonephritis by Berden et al.

Renal histopathology N (%) ^	Total N = 16	MPA N = 10	GPA N = 6
Focal	3 (19%)	1 (10%)	2 (33%)
Crescentic	6 (37%)	2 (20%)	4 (67%)
Mixed	3 (19%)	3 (30%)	0 (0%)
Sclerotic	4 (25%)	4 (40%)	0 (0%)

^Total calculated of patients who underwent renal biopsy

Pulmonary involvement on presentation was noted in 67% patients in MPA cohort and 89% patients in GPA cohort (p = 0.33) (Table 2). Patients with pulmonary involvement in MPA cohort commonly presented with hemoptysis and alveolar hemorrhage and most common imaging finding was presence of pulmonary infiltrates. 2 patients presented with respiratory failure requiring intubation. In GPA cohort, most common imaging findings included presence of pulmonary nodules and cavitation.

ENT involvement was more common in GPA cohort (89% versus 8%, p < 0.01). Biopsies from ENT area obtained in three patients with GPA and were diagnostic. Biopsy from mass from sinus in a GPA patient demonstrated granulomas necrosis and vasculitis. Ear biopsy in another patient with GPA demonstrated necrotizing mononuclear inflammation and foci of small vessel vasculitis. In a patient presenting with severe hoarseness, subglottic biopsy demonstrated presence of necrotizing vasculitis along with active inflammation.

There were no significant differences in rates of cardiac, cutaneous, and gastrointestinal involvement. In MPA cohort, one patient presented with cardiomegaly secondary to large pericardial effusion and tamponade.

With regards to ANCA status, all patients were ANCA positive. MPO antibodies were seen in 83% of MPA cohort and in 22% of patients diagnosed with GPA. All patients diagnosed with GPA tested positive for anti-PR-3 antibody, in comparison to 8% in MPA cohort (p < 0.0001). Median hemoglobin at presentation was significantly lower in MPA cohort (6.9 g/dL versus 10.4 g/dL, p = 0.03). (Table 2).

Geographic distribution of residence was metropolitan in 18 patients (86%), Micropolitan in 2 (10%) and one patient from rural area using the Rural-Urban Commuting Area Codes (RUCA) code [26]. All patients in MPA cohort were insured and only one patient was uninsured in GPA cohort. Hence, there were no differences in geographic location or insurance status between MPA and GPA cohorts.

#### Race/Ethnicity

**Fig. 2:**
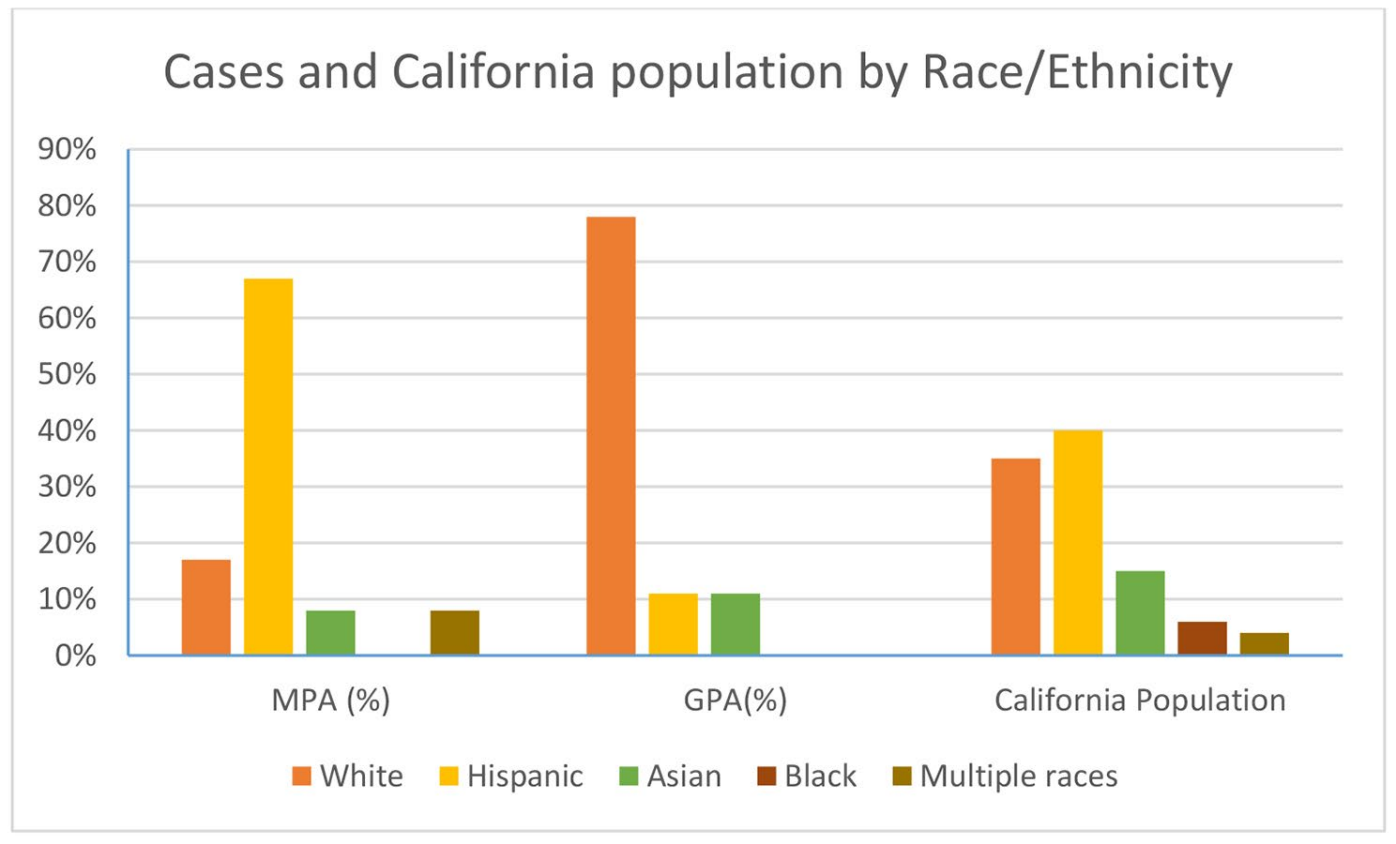
Case distribution by ethnicity and California population

57% of the cohort constituted of racial/ethnic minorities, including Hispanics (n = 9), Asians (n = 2), and multiracial (n = 1). 43% were white (n = 9). Among the Hispanic patients in our cohort, MPA was the prevailing diagnosis (8 out of 9) (Table 1). Race/ ethnicities within our cohort and comparison with California’s population are represented in Fig. 2. All Hispanic patients were positive for myeloperoxidase antibody (Table 4), including one patient diagnosed with GPA. In comparison, 7 out of 9 white patients were diagnosed with GPA and 89% were PR-3 positive. (Table 4)

**Table 4:** ANCA characteristics by ethnicity

	Hispanic (n = 9)	Asian (n = 2)	Multiracial (n = 1)	White (n = 9)	P value**
Anti-MPO positive (n, %)	9 (100%)	1 (50%)	1 (100%)	1 (11%)	< 0.05
Anti-PR-3 positive (n, %)	1 (11%)	1 (50%)	0 (0%)	8 (89%)	< 0.05

MPO = Myeloperoxidase, PR-3 = Proteinase 3, **Hispanic versus white

### Disease severity

We compared rates of intensive care unit (ICU) admission, dialysis, plasmapheresis, length of hospital stay and mortality (Table 5). 67% patients in MPA cohort and 33% in GPA cohort (p = 0.2) required admission to ICU. Dialysis was required in nine patients in our cohort (43%), of which 6 were diagnosed with MPA and 3 with GPA. Length of hospital stay was similar in both cohorts (19 days in MPA versus 18 days in GPA, p = 0.5). Plasmapheresis was used in 5 patients (24%). Rates of plasmapheresis were similar between the two cohorts (25% in MPA versus 22% in GPA; p = 1). All patients who received plasmapheresis had both pulmonary and severe renal involvement requiring dialysis. All 5 patients received either cyclophosphamide, rituximab or combination therapy (2 patients). Although none of these measures reached statistical significance, 2 deaths (n = 17%) were reported in the MPA cohort. One, in a 12-year-old Asian female presenting with severe anemia (hemoglobin 2.9 g/dL), renal failure and respiratory failure. Renal biopsy demonstrated biopsy severe parenchymal scarring and sclerotic glomeruli with remote crescents. Treatment was started with pulse intravenous methylprednisolone, intravenous cyclophosphamide 500 mg/m2 and plasmapheresis for 7 days. She developed severe pulmonary hemorrhage and alveolitis. Rituximab (375 mg/m2) was added due to continued pulmonary hemorrhage. Bronchoscopy on hospital day 29 revealed active inflammatory cells and fungal cells. This patient eventually died of Aspergillus pneumonia and sepsis. The second mortality in our cohort was a 15-year-old Hispanic female with initial presentation with hemoptysis and end-stage renal disease requiring hemodialysis. She was treated with high-dose corticosteroids and rituximab 375 mg/m2 along with plasmapheresis. This patient developed progressive respiratory distress requiring intubation and refractory hypotension.

**Table 5:** Comparison of disease severity in patients with childhood ANCA-associated vasculitis

	MPA N = 12	GPA N = 9	p-value
ICU admission (n, %)	8 (67%)	3 (33%)	0.2
Dialysis (n, %)	6 (50%)	3 (33%)	0.7
Plasmapheresis (n, %)	3 (25%)	2 (22%)	1
Mortality (n, %)	2 (17%)	0 (0%)	0.5
Median length of stay (days ,IQR)	19(13–41)	18(10–28)	0.5

IQR = Interquartile range

#### Treatment

All patients received induction treatment with high-dose pulse corticosteroids with intravenous methylprednisolone 30 mg/kg/day, with maximum dose fixed at 1000 mg/day, ranging from 3- days (Table 6). All patients received oral corticosteroids with prednisone (90%) or prednisolone (10%) after completing pulse methylprednisolone.

**Table 6:** Initial immunosuppressive therapy administered in childhood ANCA-associated vasculitis

	MPA N = 12	GPA N = 9	p- value
Pulse corticosteroids (n, %)	12 (100%)	9 (100%)	1
Corticosteroids + Cyclophosphamide (n, %)	5 (42%)	7 (78%)	0.18
Corticosteroids + Rituximab (n, %)	5 (42%)	0 (0%)	0.045
Corticosteroids + Cyclophosphamide + Rituximab (n, %)	1 (8%)	2 (22%)	0.55

15 patients (71%) received intravenous cyclophosphamide, with doses ranging from 500 to 750 mg/m2. All nine patients in GPA cohort received cyclophosphamide, either with steroids alone (n = 7, 78%) or in combination with steroids and rituximab (n = 2, 22%). Five patients (42%) in MPA cohort received cyclophosphamide in combination with steroids (Table 6), meanwhile 5 (42%) received steroids and rituximab. Use of rituximab for AAV began in 2015 in our institution. 3 patients received rituximab dosing of 375 mg per metered squared weekly x4 doses. Meanwhile 5 patients received 750 mg per metered square every 2 weeks x 2 doses. A total of 3 patients received combination therapy with cyclophosphamide and rituximab. In MPA cohort, one patient received combination therapy with cyclophosphamide and rituximab, who had a severe presentation with pulmonary-renal failure and ultimately died of sepsis and aspergillus pneumonia. 2 patients in GPA cohort received combination cyclophosphamide and rituximab- one had severe respiratory involvement, subglottic involvement with multinucleated histiocytes with necrotizing vasculitis on biopsy and another had severe pulmonary-renal involvement with necrotizing crescentic glomerulonephritis affecting 100% of glomeruli.

## Discussion

We present the largest single center study of initial presentation of childhood AAV from Central California. Given the paucity of data from our geographic area and sparse literature in pediatric patients from racial and ethnic minority backgrounds, this study provides a unique understanding of characteristics of pediatric AAV.

In our study, microscopic polyangiitis was the most frequent AAV subtype (57%) followed by granulomatosis with polyangiitis (43%). Yang et al. from China [27] and French national study by Sacri et al. [5] also found MPA as the predominant subtype in children with AAV. Studies in adults from Asia [15, 28] have also found MPA to be more common than GPA. In contrast, the largest pediatric cohort study from North America of 231 patients with in ARChiVe (A Registry for Childhood Vasculitis: e-entry) registry, found GPA to be the most common subtype [9]. Retrospective cohort study from France by Mahi et al. also reported GPA to be more frequent [10]. Differences in incidence of MPA and GPA based on geographical location have been noted in studies from Latin America as well [13]. This variability in the subtype of AAV based on geographic location is useful when evaluating children at initial presentation.

MPA cohort was slightly older at presentation at 13.7 years in comparison with other cohort studies [5, 9, 27] where median ages at presentation were around 11 years. The median age at diagnosis in GPA was 14 years and similar to other pediatric studies [5, 9]. There was a significant female preponderance, noted in other pediatric studies as well [5, 9, 10, 27]. Studies from Latin America have also observed a female predominance in adult ANCA associated vasculitis [14, 29]. Meanwhile in several adult studies, AAV has been noted to be more frequent in males than in females [7, 30].

In our study over half of the cases were diagnosed during 2015–2018. Reasons for this are unclear but we hypothesize that environmental factors implicated in pathogenesis of AAV including silica in soil, dust storms and farming could have contributed [31, 32]. Interestingly, since 2015, there has been a substantial increase in the incidence of coccidiomycosis, which is acquired by inhalation of fungal spores in the soil and is endemic to Central California [33]. Coccidiomycosis outbreaks have been linked to disturbances in the soil such as strong winds or during agricultural activity which is common in Central California [34]. Future studies are required to investigate environmental factors unique to our region, which may contribute to pathogenesis of AAV.

With regards to organ involvement, ENT involvement was frequent in GPA and similar to prior literature [5, 9, 10]. We observed significantly high rates of renal involvement (90%). There were no statistically significant differences in renal involvement between MPA (100%) and GPA (78%). Yang et al. also found frequent renal involvement (93.75%) in their cohort [27]. Meanwhile, rates of renal involvement were 75% and 88% respectively in ARChiVe and French national studies [5, 9].

Renal biopsies frequently demonstrated sclerotic and mixed involvement in MPA, while crescentic involvement was most frequent in GPA. Differences in long-term renal outcomes have been observed depending on renal histopathological patterns with focal class having most favorable outcome, meanwhile sclerotic subtype is associated with poor long-term renal outcomes [27, 35, 36]. Mahi et al. observed a higher risk of relapse in patients with mixed form [10]. Long term outcomes in crescentic class has been variable [25].

In keeping with the severity of histopathologic findings, 43% of our cohort required dialysis at initial presentation. This is higher than previously reported pediatric studies. In ARChiVe study, 25% patients required dialysis [9] and 16% in study by Morishita et al. [37]. Mahi et al. reported 19% of their cohort were on dialysis [10]. Yang et al. reported ESRD in 16% of their cohort at time of diagnosis [27]. Elevated baseline creatinine and requirement for dialysis has been identified as an independent risk factor for mortality in AAV [25, 38, 39]. We observed a consistent trend towards higher rates of ICU admission and need for dialysis in MPA. Mahi et al. also observed more severe kidney injury in their MPA cohort compared to other AAV subtypes [10].

Interestingly, shorter duration of symptoms was reported at disease onset in comparison to other studies [5, 9]. Cause for this is unclear, but may be attributed to severe disease at onset, under-recognition of symptoms of AAV in children and language barriers.

Racial/ethnic minorities constituted majority of our cohort and 43% were Hispanic. Majority resided in Fresno, Kern and Tulare counties, which have a Hispanic majority population of 54.7%, 56.1% and 66.7% respectively. Similar to adult studies [40, 41], pediatric studies in AAV include predominantly white patients [4, 5, 9, 42]. ARChiVe study had predominantly white patients and only 5% were Hispanic [9]. Largest study to date assessing disease outcomes in pediatric ANCA vasculitis, included 3 children of Hispanic ethnicity out of a cohort of 105 [37]. Despite Hispanic population constituting 18.9% of United States [43] and 40% of California’s population [22], there is notable underrepresentation of Hispanic children in AAV studies. In our study, Hispanic children were more frequently diagnosed with microscopic polyangiitis. Similarly, in ARChiVe study, MPA cohort had a higher proportion of Hispanic subjects [9]. All Hispanic patients were MPO positive. Recent study from Southern California also found adult Hispanic patients were more often diagnosed with MPA and had greater MPO positivity [17]. Similar observations were found in study from Peru [14]. Hence, it is important to consider differences in clinical presentation of AAV in different ethnic groups.

Pediatric studies comparing disease presentation and outcomes based on ethnicity are lacking in AAV. While our current study was underpowered to evaluate these differences, race/ethnicity and various socioeconomic factors are known to correlate with disease severity and progression in lupus and other autoimmune diseases [44, 45]. Sreih et al. found more severe disease and higher damage indices in Hispanics with AAV, including renal involvement and dialysis requirement in comparison to Caucasians [16]. Lee et al. reported more frequent flares, end-stage renal disease, severe pulmonary manifestations and ICU admissions in Hispanics from Southern California [17]. Future studies are needed to identify role of various social determinants of health including race/ ethnicity as potential factors in outcomes of pediatric ANCA associated vasculitis.

Treatment of pediatric AAV largely relies on data extrapolated from adult, even though long term effects of these medications on a developing immune system continue to be studied [46]. Use of corticosteroids at treatment induction was universal, similar to other studies [5, 9, 10, 27]. A significant proportion of patients (71%) received cyclophosphamide for induction treatment. Rituximab was utilized in more than a third of our cohort, with greater use in MPA. In French study by Mahi et al., 37% received rituximab and 17.4% received a combination of cyclophosphamide and rituximab [10]. Yang et al. reported use of combination therapy in 14.5%, with most having MPA [27]. In early outcomes study by Morishita et al., 70% received cyclophosphamide for induction, 13% received rituximab and less than 4% received both [37]. In French national study by Sacri et al.,66% received cyclophosphamide and 14% received rituximab [5]. In ARChiVe study, 76% received cyclophosphamide and 12% were treated with rituximab [9]. Randomized control trials in adults have demonstrated non-inferiority of rituximab in comparison to cyclophosphamide [47, 48]. American College of Rheumatology/Vasculitis Foundation guidelines conditionally recommend treatment with rituximab over cyclophosphamide for remission induction for severe disease [49]. CARRA consensus treatment plans for severe pediatric AAV include rituximab in primary remission induction treatment [46]. 3 patients (14%) in our cohort received combination treatment with cyclophosphamide and rituximab, of which 2 had severe renal disease with serum creatinine > 6 mg/dL at presentation. KDIGO guidelines support considering use of combination of rituximab and cyclophosphamide in patients with severe kidney disease with markedly reduced GFR, including serum creatinine > 4 mg/dl [50]. Rituximab is being increasingly used for treatment induction, while the combination with cyclophosphamide remains uncommon. Long-term effects of rituximab, such as hypogammaglobinemia and risk of infections need to be further studied in pediatric population.

All 5 patients who received plasmapheresis had severe pulmonary and renal involvement and 2 deaths were observed. American College of Rheumatology/ Vasculitis Foundation guidelines conditionally recommended against routine addition of plasma exchange to remission induction therapy [49]. Since majority of our study period included years prior to release of the standardized treatment guidelines, there is likely variability in treatment regimens and medication dosages. We hope future data will be better characterized.

Our study has several limitations which include its retrospective design and information was extracted from available medical record documentation. Due to limited availability of prior outpatient EMR records scanned into current electronic record system and lack of Pediatric Vasculitis Activity Scores (PVAS), we are unable to study rates of remission, disease relapse rates and long term outcomes. Furthermore, due to rarity of AAV in children, we had a relatively small number of patients. It is also possible to have underreported cases due to retrospective nature of the study. Also this is single center study and findings may not be generalizable to other locations. Despite these limitations, our study is the largest single center study of AAV of children in California and the first to report the characteristics in childhood ANCA vasculitis in Central California.

## Conclusion

We report the largest single center study of initial presentation of AAV from a tertiary care children’s hospital in California. Microscopic polyangiitis was the most frequent AAV subtype followed by granulomatosis with polyangiitis. We observed a higher proportion of racial/ ethnic minority patients in our cohort with female preponderance, shorter duration of symptoms at disease onset and high rates of renal involvement, especially in the MPA cohort. We observed more frequent MPO positivity in Hispanic children. Trend towards higher rates of ICU admission and need for dialysis were observed in patient with MPA. Corticosteroid use was universal and patients frequently received induction treatment with cyclophosphamide. More than one third of our cohort received rituximab, either alone or in combination with cyclophosphamide. Patients with MPA received rituximab more frequently. Mortality in MPA cohort occurred due to infection (Aspergillus pneumonia) and pulmonary hemorrhage. This study highlights the need for further research to understand the impact of race/ ethnicity on pediatric AAV presentation, disease activity, and outcomes. Further prospective studies are needed to understand differences in AAV between diverse racial-ethnic groups residing in the same geographic region.

## Data Availability

Corresponding author can provide the datasets used and analyzed during the current study, on reasonable request.

## Abbreviations

AAV: ANCA- associated vasculitis
ANCA: Antineutrophil cytoplasmic antibody
ARChiVe: A Registry for Childhood Vasculitis: e-entry
EGPA: Eosinophilic granulomatosis with polyangiitis
ENT: Ear, nose, and throat
EULAR: European Alliance of Associations for Rheumatology
GPA: Granulomatosis with polyangiitis
ICU: Intensive care unit
IV: Intravenous
IQR: Interquartile range
MPA: Microscopic polyangitis
MPO: Myeloperoxidase
PR3: Proteinase 3
PReS: Pediatric Rheumatology European Society
PRINTO: Pediatric Rheumatology International Trials Organization
RUCA: Rural-Urban Commuting Area Codes
